# Variable effects of nicotine, anabasine, and their interactions on parasitized bumble bees

**DOI:** 10.12688/f1000research.6870.2

**Published:** 2015-12-16

**Authors:** Lukas P. Thorburn, Lynn S. Adler, Rebecca E. Irwin, Evan C. Palmer-Young

**Affiliations:** 1 Department of Biology, University of Massachusetts at Amherst, Amherst, Massachusetts, USA; 2 Department of Biology, Dartmouth College Hanover, New Hampshire, USA; 3 Department of Applied Ecology, North Carolina State University, Raleigh, North Carolina, USA

**Keywords:** Bumble bee, Bombus impatiens, parasites, Crithidia bombi, plant secondary metabolites, nicotine, alkaloids, tritrophic interactions

## Abstract

Secondary metabolites in floral nectar have been shown to reduce parasite load in two common bumble bee species. Previous studies on the effects of nectar secondary metabolites on parasitized bees have focused on single compounds in isolation; however, in nature, bees are simultaneously exposed to multiple compounds. We tested for interactions between the effects of two alkaloids found in the nectar of *Nicotiana* spp. plants, nicotine and anabasine, on parasite load and mortality in bumble bees ( *Bombus impatiens*) infected with the intestinal parasite *Crithidia bombi*. Adult worker bees inoculated with *C. bombi* were fed nicotine and anabasine diet treatments in a factorial design, resulting in four nectar treatment combinations:  2 ppm nicotine, 5 ppm anabasine, 2ppm nicotine and 5 ppm anabasine together, or a control alkaloid-free solution. We conducted the experiment twice: first, with bees incubated under variable environmental conditions (‘Variable’; temperatures varied from 10-35°C with ambient lighting); and second, under carefully controlled environmental conditions (‘Stable’; 27°C incubator, constant darkness). In ‘Variable’, each alkaloid alone significantly decreased parasite loads, but this effect was not realized with the alkaloids in combination, suggesting an antagonistic interaction. Nicotine but not anabasine significantly increased mortality, and the two compounds had no interactive effects on mortality. In ‘Stable’, nicotine significantly increased parasite loads, the opposite of its effect in ‘Variable’. While not significant, the relationship between anabasine and parasite loads was also positive. Interactive effects between the two alkaloids on parasite load were non-significant, but the pattern of antagonistic interaction was similar to that in the variable experiment. Neither alkaloid, nor their interaction, significantly affected mortality under controlled conditions. Our results do not indicate synergy between *Nicotiana* nectar alkaloids; however, they do suggest a complex interaction between secondary metabolites, parasites, and environmental variables, in which secondary metabolites can be either toxic or medicinal depending on context.

## Introduction

Throughout the past two decades, many wild and managed bee species have experienced severe declines ( Allen-Wardell *et al.*, 1998; Cameron *et al.*, 2011; Potts *et al.*, 2010). In many cases of bee decline, parasitism has been implicated as a potential cause (reviewed in Goulson *et al.*, 2015 and Potts *et al.*, 2010). Secondary metabolites – plant compounds that do not play a role in the plant’s primary metabolism – frequently have antimicrobial properties ( Schmidt *et al.*, 2012), and could offer a means of natural parasite control. Secondary metabolites are found in the floral nectar of many plant species ( Heil, 2011).

The effects of secondary metabolites on insects, including bees and other pollinators, are context-dependent. A wide range of secondary metabolites, including terpenes, alkaloids, and phenolics, are toxic to insects ( Detzel & Wink, 1993; Kumrungsee *et al.*, 2014; Raffa *et al.*, 1985; Singaravelan *et al.*, 2006; Wink & Theile, 2002). Interaction with other stressors, such as infection or climatic stress, can exacerbate these toxic effects ( Goulson *et al.*, 2015; Holmstrup *et al.*, 2010; Köhler *et al.*, 2012a). However, under some circumstances, the antimicrobial properties of secondary metabolites can provide health benefits to infected insects. Insects have been shown to self-medicate with secondary metabolites in response to parasite infection (reviewed in Abbott, 2014). For example, *Grammia incorrupta* (wooly bear) caterpillars exhibited self-medication behavior in response to tachinid fly parasitism by increasing their consumption of pyrrolizidine alkaloids, which decreased the survival of unparasitized caterpillars but increased the survival of parasitized caterpillars ( Singer *et al.*, 2009).

Several recent studies have indicated that plant secondary metabolites, including those found in nectar, can benefit infected pollinators as well. Honey bees self-medicated in response to parasitism through increased foraging for resins, which are used in hive construction and have antimicrobial properties ( Simone-Finstrom & Spivak, 2012), and through preferentially feeding on certain types of honey, such as sunflower honey, which reduced pathogen load ( Gherman *et al.*, 2014). Bumble bees ( *Bombus terrestris*) infected with the intestinal parasite *Crithidia bombi* displayed increased preference for nicotine-containing artificial nectar, which also reduced parasite load ( Baracchi *et al.*, 2015). In another bumble bee species ( *B. impatiens*), consumption of the alkaloid gelsemine significantly reduced *C. bombi* infection intensity ( Manson *et al.*, 2010), and in a separate study, four other nectar secondary compounds had significant medicinal effects, with an additional four compounds causing non-significant decreases in infection severity ( Richardson *et al.*, 2015a).

Previous studies of the effects of nectar secondary metabolites on pollinators have focused primarily on single compounds in isolation. Under natural conditions, however, pollinators would likely encounter several compounds at once, since many plant species produce multiple secondary metabolites. For example, many *Nicotiana* species contain both nicotine and anabasine in nectar ( Adler *et al.*, 2012), and *Chelone glabra* contains the iridoid glycocides aucubin and catalpol in nectar ( Richardson *et al.*, 2015b). This raises the possibility of interactions between nectar secondary metabolites.

Synergistic interactions between secondary metabolites from other plant tissues are well established. Among herbivores, the iridoid glycosides aucubin and catalpol had synergistic effects on the survival of common buckeye ( *Junonia coenia* Hübner) caterpillars that specialize on plants with these compounds; caterpillars that consumed both iridoid glycosides had an increased rate of survival relative to caterpillars that consumed either glycoside alone ( Richards *et al.*, 2012). Amides in plants in the *Piper* genus had synergistic deterrent effects on herbivorous ants, while the same compounds were neutral or attractive in isolation ( Dyer *et al.*, 2003). Synergy between secondary metabolites can also alter antimicrobial effects. Carvacrol and thymol, for example, inhibited the growth of the bacterium *Listeria innocua* more effectively in combination than alone ( García-García *et al.*, 2011). Carvacrol was also more effective against the bacterium *Vibrio cholerae* when combined with cymene, although cymene alone had no antimicrobial activity ( Rattanachaikunsopon & Phumkhachorn, 2010).

Antagonism between secondary metabolites has also been demonstrated. The deterrent effect of the amide piperine on the hemipteran *Sibaria englemani* is significantly reduced when piperine is combined with the amide piplartine, although piplartine alone had no effect on *S. englemani* feeding preference ( Whitehead & Bowers, 2014). The linear furanocoumarins psoralen, bergapten, and xanthotoxin exhibited antagonistic interactions in their effects on insect mortality; the toxicity of psoralen combined with either or both of the other two compounds was significantly lower than would be predicted based on their toxicities in isolation ( Diawara *et al.*, 1993). If similar interactions, either synergistic or antagonistic, are present between secondary metabolites in nectar, they could exacerbate or ameliorate the effects of single compounds found in previous studies.

To evaluate interactions between secondary metabolites from the nectar of a single plant, we tested the effects of nicotine and anabasine alone and in combination on bumble bee resistance to the gut parasite *Crithidia bombi*. Nicotine and anabasine co-occur in the nectar of several species in the genus *Nicotiana*, which includes cultivated tobacco ( *Nicotiana tabacum*) as well as several ornamental species ( Adler *et al.*, 2012). The effects of nicotine and anabasine in combination on bee disease have not previously been studied. In addition, this is the first study to our knowledge that explicitly tests for interactive effects of multiple secondary compounds on bumble bee disease.

We tested the effects of these compounds in two environmental contexts, variable and controlled conditions. Bumble bees in the wild encounter a wide range of environmental conditions, which could alter the effects of diet and parasitism. In general, temperature can decrease tolerance to environmental toxins, including secondary metabolites ( Holmstrup *et al.*, 2010), and exert unpredictable effects on insect-parasite interactions through modulation of host survival, host immune function, and parasite viability ( Thomas & Blanford, 2003). Variable temperatures impose exceptional energetic costs on bumble bees by forcing them to actively regulate body temperature in order to fly ( Heinrich, 1972). These costs might create caloric deficits that increase parasite virulence in *Bombus* ( Brown *et al.*, 2000). Alternatively, heightened energy needs could lead to increased consumption of plant foods, thereby elevating exposure to secondary metabolites. Globally, responses to environmental variability have implications for conservation: Bumble bee species with narrow climatic ranges are particularly vulnerable to decline ( Williams *et al.*, 2007; Williams *et al.*, 2009), and projected climate change may further restrict these species’ distributions through increases in mean temperature and the frequency of extreme events ( Diffenbaugh & Field, 2013).

## Methods

### Study system

*Bombus impatiens* is the most common bumble bee species in eastern North America, with a range extending from Ontario and Maine to southern Florida ( Balaban *et al.*, 2014). It is an important pollinator in agriculture, and commercial distribution of *B. impatiens* is becoming increasingly common ( Colla *et al.*, 2006).

*Crithidia bombi* is a common trypanosome parasite of bumble bees in Europe and North America ( Colla *et al.*, 2006; Lipa & Triggiani, 1988). Its range has been expanding within North America and into parts of South America, potentially due to spillover from commercial to wild bumble bee populations ( Colla *et al.*, 2006; Schmid-Hempel *et al.*, 2014; but see Whitehorn *et al.*, 2013). *C. bombi* is known to increase mortality in bumble bees under food stress conditions ( Brown *et al.*, 2000), and to reduce bumble bee foraging rate ( Otterstatter & Thomson, 2006).

Nicotine is an agonist of the nicotinic acetylcholine receptor (nAChR), and therefore acts as both a stimulant drug and a toxin to many organisms ( Benowitz, 1998). Nicotine is toxic to many insects, and has been historically used as an insecticide ( Ujváry, 1999). Honey bees are deterred by nicotine in nectar ( Köhler *et al.*, 2012b), and both honey bees ( Köhler *et al.*, 2012b; Singaravelan *et al.*, 2006) and bumble bees ( Baracchi *et al.*, 2015) are adversely affected by nicotine consumption when they are not infected by parasites. However, nicotine also has antimicrobial properties ( Pavia *et al.*, 2000), and recent studies have suggested that it can reduce parasite load in bumble bees infected with *C. bombi* ( Baracchi *et al.*, 2015; Richardson *et al.*, 2015a), and may improve survival of diseased honey bee colonies ( Köhler *et al.*, 2012b). Anabasine, like nicotine, is a nAChR agonist, and has been used as an insecticide ( MacBean, 2012). The behavioral effects of anabasine are similar to those of nicotine, although anabasine, unlike nicotine, does not have addictive effects ( Caine *et al.*, 2014). Anabasine in nectar deterred honey bees ( Singaravelan *et al.*, 2005), and reduced *C. bombi* load in infected bumble bees ( Richardson *et al.*, 2015a).

### Secondary compound treatments

We inoculated bumble bees with *C. bombi*, and assessed the differences in pathogen load and mortality between adult bees fed nicotine (yes/no) and anabasine (yes/no) in a factorial design, resulting in four diet treatments: 2 ppm nicotine, 5 ppm anabasine, 2 ppm nicotine and 5 ppm anabasine together, or a control alkaloid-free solution. All diet treatments also contained 30% sucrose in distilled water. Chemicals ((-)-nicotine, cat. no. N3876; (+/-)-anabasine, cat. no. 284599) were purchased from Sigma-Aldrich (St. Louis, MO). Alkaloid concentrations were chosen to mimic the highest concentrations that would be found in *Nicotiana* nectar under natural conditions ( Adler *et al.*, 2006; Tadmor-Melamed *et al.*, 2004).

### Rearing conditions

We conducted two experiments under different environmental conditions. The first experiment (‘Variable’, conducted 26 February 2014 to 20 March 2014, Dataset 1) had a smaller sample size (n = 178 bees) and variable environmental conditions. In ‘Variable’, experimental bees and pupae were kept on the lab bench in a room where temperatures fluctuated between 10 and 35°C. This fluctuation was the result of a building steam leak. During most of the daytime when experimenters were present, the temperature was near 35°C, although on several weekends the heating system was shut down entirely for repairs, and temperatures as low as 10°C were reached. Bees in ‘Variable’ were also exposed to everyday stimuli and ambient fluorescent and window lighting (approximately 12 h photoperiod).

The second experiment (‘Stable’, conducted 20 May 2014 to 14 July 2014, Dataset 2) had a larger sample size (n = 339 bees) and carefully controlled environmental conditions (see sample sizes in Table S1). In ‘Stable’, experimental bees as well as pupae were kept in an incubator at 27°C in constant darkness to reduce mortality and more closely mimic conditions in a bumble bee hive.

Experimental bees were obtained from pupal clumps of commercial *B. impatiens* (Biobest, Leamington, Ontario, Canada). Pupal clumps were removed from colonies weekly and kept in 500 mL plastic containers, with each container containing the pupal clumps from a single colony that were collected on a specific date. In ‘Variable’, pupal clumps were incubated on the lab bench for the majority of the experiment until 2 d before the emergence of the final experimental bees, at which time the clumps were moved to a 30°C incubator (Percival Scientific, Perry, IA) due to excessive pre-experiment mortality under the variable lab conditions. Hence, all bees would have spent the majority of the pupation period under variable conditions. In ‘Stable’, pupal clumps were incubated at 27°C in darkness throughout the experiment. In both experiments, callow bees (newly emerged worker bees less than one day old) were collected upon emergence from pupal clumps. They were weighed and their mass at emergence, date of emergence, and colony of origin were recorded. Bees were then isolated in individual 20 mL vials. The lid of each vial was equipped with a 2 mL microcentrifuge tube with a cotton wick containing 500 μL artificial nectar (30% sucrose solution). Each day, bees were transferred to clean vials and given 500 μL fresh artificial nectar and a 10 mg piece of multifloral pollen (Koppert Biological Systems, Howell, MI) on which they fed *ad libitum*. For two days after emergence, bees were fed pollen and control nectar (30% sucrose solution), then were inoculated with *C. bombi*. They were starved for several hours to ensure that they would consume the inoculum, then fed 10 μL of *C. bombi* inoculum containing 6,000 *C. bombi* cells (see below). Following inoculation, bees were fed the appropriate nectar treatment and pollen *ad libitum* for 7 days. Bees were assigned systematically to secondary compound treatments in blocks of four, such that each block contained a bee in each treatment.

### Inoculation

To inoculate experimental bees, inoculum ( *C. bombi* cells in sucrose solution) was prepared from the gut tracts of bees taken from colonies infected with *C. bombi*. These colonies were obtained from the same supplier as the experimental colonies, and were infected with *C. bombi* from wild bees collected in Amherst, Massachusetts (September 2013). Infected bees were dissected and their gut tracts were macerated with a plastic pestle in microcentrifuge tubes containing 300 μL distilled water. Samples were incubated for 5 hours at room temperature to allow gut tissue to settle. *C. bombi* cell density was then assessed using a hemocytometer, and inoculum was prepared from the supernatant of the samples with sufficient concentrations of *C. bombi* cells. The supernatant was diluted to a concentration of 1200 cells/μL and had an equal volume of 50% sucrose solution added to result in a 25% sucrose solution. Each bee was fed 10 μL of inoculum, containing 6,000 *C. bombi* cells, using a 20 μL micropipette.

### Bumble bee dissection and parasite quantification

Seven days after inoculation, bees were dissected to assess parasite loads. Gut tracts were extracted and crushed with a pestle in microcentrifuge tubes containing 300 μL distilled water. Samples were allowed to sit for 5 hours to allow gut tissue to settle. *C. bombi* cell concentrations in the gut extract were measured using a hemocytometer. *C. bombi* cells were counted in five cells of the hemocytometer and summed (0.004 µL each; 0.02 µL total).

### Consumption experiment

To determine the effects of temperature on nectar consumption, we measured 24 h nectar consumption of bees incubated at 2 different temperatures, 27 or 33°C. Bees were removed from their natal colonies and starved in snap-cap vials 2 h before the experiment began. To begin the experiment, bees were given access to a 2 mL microcentrifuge feeding tube for 24 h. The feeding tube was filled with 1 mL artificial nectar (30% w/w sucrose) and punctured in the center of the lid with a 0.8 mm diameter sewing needle to allow bees access to the nectar. The upper half of the feeding tube was then inserted into a hole punched in the vial cap. Vials were incubated on their sides at a 10° angle in constant darkness. The feeding tube was weighed at the beginning and end of the 24 h experiment to estimate nectar consumption. The average mass loss of 6 control tubes, which were incubated identically in vials without bees, was subtracted from each bee’s estimated consumption to correct for leakage and evaporation. Bees were weighed post-experiment to allow use of bee mass as a model covariate. At each temperature, we ran 3 one-day trials of 24 bees (12 from each of 2 colonies), alternating between days of 27 and 33°C.

### Statistics

Data were analyzed using R version 3.2.1 for Windows ( R Core Team, 2014).

### Mortality data

For ‘Variable’, for which exact dates of death were not recorded, mortality was analyzed using a generalized linear mixed model with binomial error distribution ( Pinheiro *et al.*, 2015). Probability of death was used as the response variable with nicotine treatment, anabasine treatment, and their interaction as predictor variables. Bee colony was included as a fixed predictor, and date of inoculation was included as a random factor. Wald tests ( Lesnoff & Lancelot, 2012) were used to test the marginal significance of individual predictor variables (see Supplementary material script 1). Mortality data for ‘Stable’, in which we recorded time from inoculation to death to the nearest day, were analyzed using a Cox proportional hazards mixed-effects model ( Therneau, 2015). Death hazard rate was used as the response variable; nicotine, anabasine, and their interaction as predictor variables; colony as a fixed predictor; and date of inoculation as a random factor (see Supplementary material script 3). To facilitate direct comparison between results of ‘Variable’ and ‘Stable’, the results of ‘Stable’ were also analyzed with the same binomial mixed model used for the ‘Variable’ experiment.

### Parasite load

Parasite counts were found to best fit the log-normal distribution and were analyzed using generalized linear mixed models ( Bates *et al.*, 2015) with penalized quasi-likelihood parameter estimation ( Venables & Ripley, 2002). Parasite counts were (x+1)-transformed for use as the response variable. Nicotine, anabasine, and their interaction were used as predictor variables. Bee colony was included as a fixed predictor, mass (at emergence from pupations) as a model covariate, and date of inoculation as a random factor. Marginal significance of individual terms was evaluated using Wald tests ( Lesnoff & Lancelot, 2012). Code for analysis is given in Supplementary material script 2 (‘Variable’ experiment) and Supplementary material script 4 (‘Stable’ experiment).

### Consumption experiment

Nectar consumption was analyzed using an analysis of variance with 24 h net nectar consumption as the response variable, temperature and colony as fixed predictors, and bee mass as a covariate. We excluded bees that died during the 24 h trial.

## Results

Data for ‘Variable’ experiment Abbreviations: bee.ID—unique number assigned to each experimental bee; source.colony—colony of origin; treatment—letter corresponding to one of four diet treatments: “C” = control, “N” = nicotine (2 ppm), “A”= anabasine (5 ppm), “together” = nicotine (2 ppm) with anabasine (5 ppm); Nicotine.treatment—binary variable for diet treatment indicating “0” for no nicotine or “1” for 2 ppm nicotine; Anabasine.treatment—binary variable for diet treatment indicating “0” for no anabasine or “1” for 5 ppm anabasine; mass—mass of bee at time of emergence from pupal clump; inoculation.date—date of inoculation; inoculated—binary variable indicating whether bee was successfully inoculated (“1”) or not (“0”); dead.before.dissection—binary variable indicating whether bee died (“1”) or survived (“0”) until the time of dissection at 7 days; dissection.count—number of *C. bombi* cells counted in 0.02 µL gut extract. Click here for additional data file. Copyright: © 2015 Thorburn LP et al. 2015 Data associated with the article are available under the terms of the Creative Commons Zero "No rights reserved" data waiver (CC0 1.0 Public domain dedication).

Data for ‘Stable’ experiment Abbreviations: bee—unique number assigned to each experimental bee; colony—colony of origin; treatment—describes one of four diet treatments: “Control” = control; “Nicotine” = nicotine (2 ppm), “Anabasine”= anabasine (5 ppm), “Nic + Ana” = nicotine (2 ppm) with anabasine (5 ppm); Inoc.Date—date of inoculation; Nicotine—column denoting whether nectar treatment contained (“Yes”) or did not contain (“No”) 2 ppm nicotine; Anabasine—column denoting whether nectar treatment contained (“Yes”) or did not contain (“No”) 5 ppm anabasine; nicotine—binary variable for diet treatment indicating “0” for no nicotine or “1” for 2 ppm nicotine; anabasine—binary variable for diet treatment indicating “0” for no anabasine or “1” for 5 ppm anabasine; mass—mass of bee at time of emergence from pupal clump; Time.To.Death—number of days from inoculation to death, with negative numbers denoting excluded bees that died before inoculation or escaped before dissection; Dead.Binary—binary variable indicating whether bee died (“1”) or survived (“0”) until the time of dissection at 7 days; count—number of *C. bombi* cells counted in 0.02 µL gut extract. Click here for additional data file. Copyright: © 2015 Thorburn LP et al. 2015 Data associated with the article are available under the terms of the Creative Commons Zero "No rights reserved" data waiver (CC0 1.0 Public domain dedication).

### ‘Variable’

**Figure 1.**
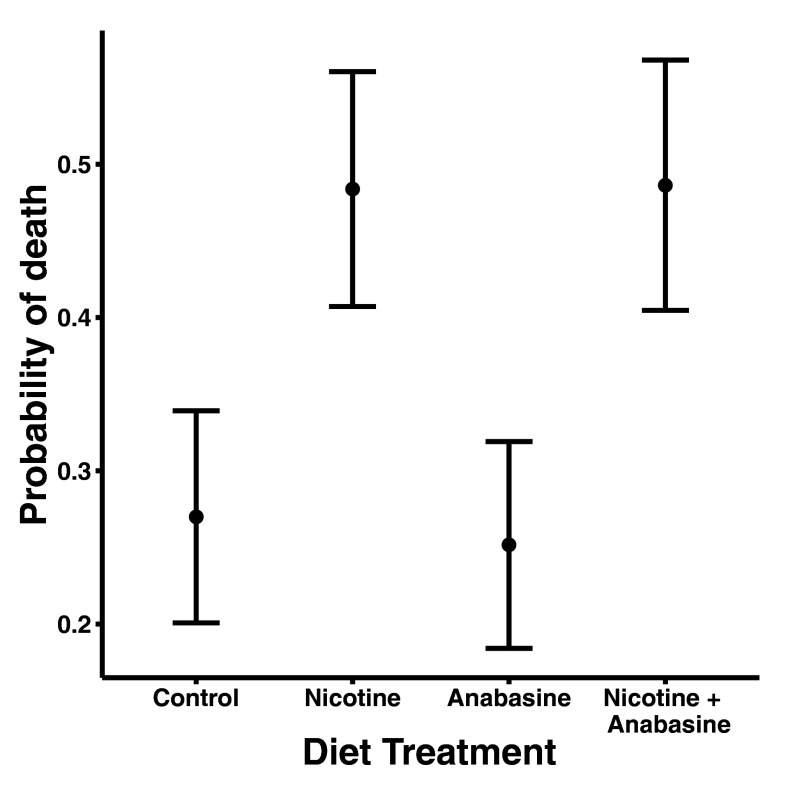
Effects of nicotine and anabasine on mortality in ‘Variable’ experiment. Points show adjusted mean probability of death in each treatment group. Error bars represent ±1 standard error. Sample sizes: n=45 (Control), n=46 (Nicotine), n=46 (Anabasine), n=41 (Nicotine + Anabasine).

In variable temperature conditions, the nicotine treatment significantly increased mortality ( Table 1). Nearly half of bees fed nicotine-containing nectar died within 7 days of inoculation, which was nearly double the frequency of death in treatments without nicotine ( Figure 1). Anabasine did not affect mortality, and there was no significant interaction between the two alkaloid treatments ( Figure 1, Table 1).

**Table 1.** Effects of nicotine and anabasine consumption on mortality in ‘Variable’ experiment. Table shows binomial mixed model results of χ2 tests for effects of predictor variables on probability of death during the 7 d experiment.

Source	χ2	Df	P
Nicotine	4.1749	1	0.041
Anabasine	0.0374	1	0.85
Nicotine*Anabasine	0.0256	1	0.87
Colony	0.911	4	0.92

**Figure 2.**
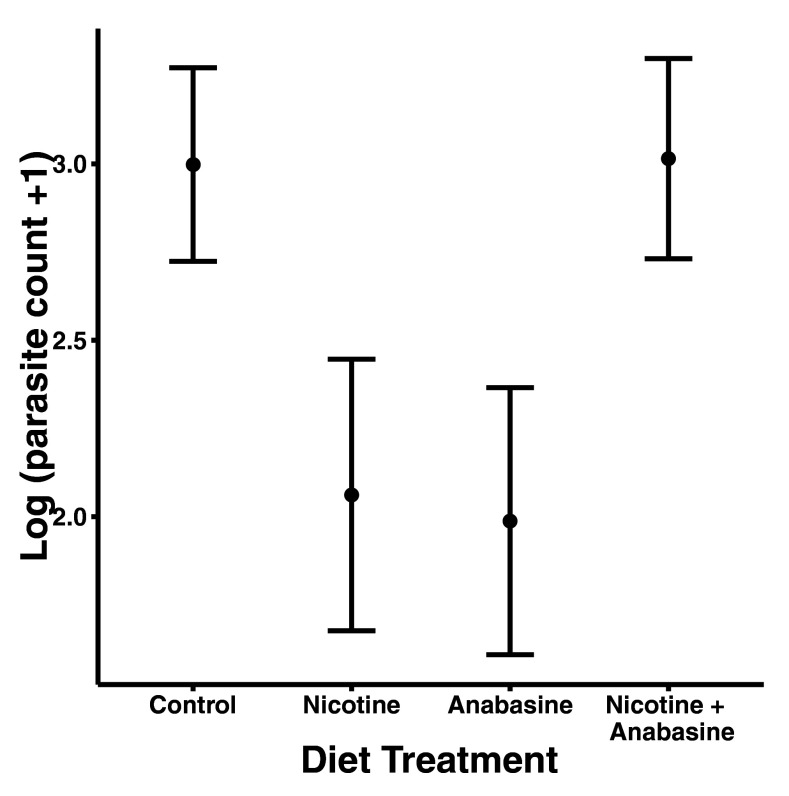
Effects of nicotine and anabasine on parasite load in ‘Variable’ experiment. Points show adjusted mean parasite count in each treatment group. Error bars represent ±1 standard error. Sample sizes: n=31 (Control), n=24 (Nicotine), n=33 (Anabasine), n=20 (Nicotine + Anabasine).

Nicotine (linear model β = -1.01 ± 0.295 standard error) and anabasine (β = -0.94 ± 0.31 S.E.) each significantly decreased parasite loads. However, nicotine and anabasine displayed antagonistic effects (Nicotine * Anabasine β = 1.96 ± 0.44 S.E.), such that bees consuming both alkaloids did not realize the medicinal effects of either compound ( Figure 2, Table 2). Parasite load had a significant negative relationship with bee mass (β = -11.17 ± 3.60 S.E., Table 2), indicating that larger bees had lower parasite loads after controlling for other factors.

**Table 2.** Effects of nicotine and anabasine on parasite loads in ‘Variable’ experiment. Results of Wald tests for marginal significance of terms in a generalized linear mixed model with penalized quasi-likelihood parameter estimation. “Mass” refers to bee mass at time of emergence.

Source	χ2	Df	P
Nicotine	10.054	1	0.0025
Anabasine	12.843	1	<0.001
Nicotine*Anabasine	22.045	1	<0.001
Colony	15.48	4	0.0038
Mass	10.517	1	0.0012

### ‘Stable’

**Figure 3.**
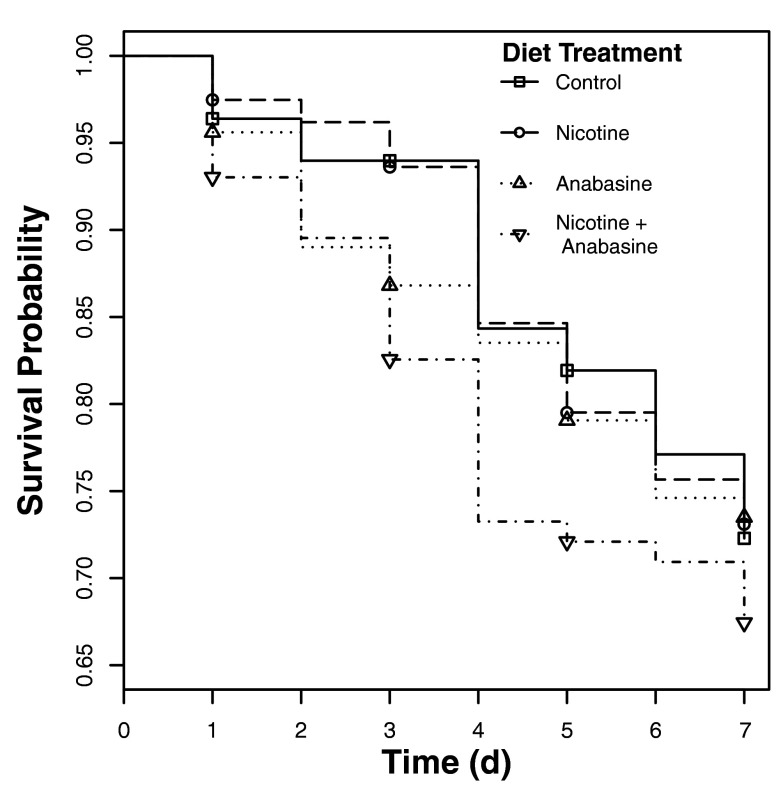
Effects of nicotine and anabasine on mortality in ‘Stable’ experiment. Lines show survival curves for bees each treatment group. There were no significant effects of diet treatments on survival. Sample sizes: n=83 (Control), n=79 (Nicotine), n=91 (Anabasine), n=86 (Nicotine + Anabasine).

**Figure 4.**
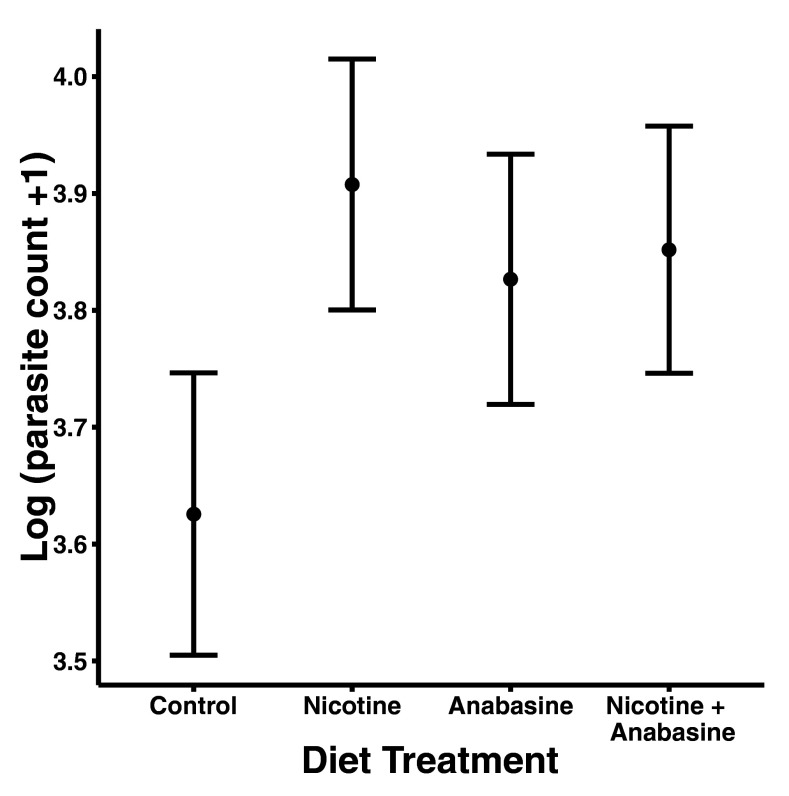
Effects of nicotine and anabasine on parasite load in ‘Stable’ experiment. Points show adjusted mean parasite count in each treatment group. Error bars represent ±1 standard error. Sample sizes: n=60 (Control), n=59 (Nicotine), n=66 (Anabasine), n=61 (Nicotine + Anabasine).

Under controlled conditions (27°C with constant darkness), neither alkaloid nor their interaction significantly affected mortality ( Figure 3, Table 3, Table S2). Although the death hazard ratio was lowest in the Control group (estimate = 0.82, 95% CI = (0.56, 1.19)) and highest in the group that received both alkaloids (estimate = 1.19, 95% CI = (0.90, 1.78)), this difference did not approach statistical significance (pairwise comparison: z = 1.49, *P* = 0.45). However, nicotine significantly *increased* parasite loads (β = 0.28 ± 0.12 S.E., Table 4), while the effects of anabasine (β = 0.20 ± 0.12 S.E.) were also positive but not significant ( Figure 4, Table 4). This was the opposite result of that observed in ‘Variable’, in which alkaloid ingestion decreased the severity of *Crithidia* infection. Although much weaker than in ‘Variable’, we found the same pattern of antagonistic interaction between the two alkaloids (Nicotine * Anabasine β = -0.26 ± 0.16 S.E., Figure 4), indicating that the deleterious effects of each compound were reduced in bees consuming the nicotine + anabasine combination ( Figure 4). However, this interaction was not statistically significant ( Table 4). Overall parasite loads in ‘Stable’ were much higher, with median parasite counts (37.5 cells * 0.02 µL ^-1^) more than triple those observed in ‘Variable’ (11.0 cells * 0.02 µL ^-1^). As in ‘Variable’, there was a significant negative relationship between mass at emergence and parasite count (β = -3.41 ± 1.32 S.E., Table 4). Average bee mass in ‘Stable’ (121 mg) was significantly lower than in ‘Variable’ (160 mg) (t(290) = -9.17, *P*<0.001), possibly reflecting elevated pre-experiment mortality of smaller bees under the fluctuating conditions of ‘Variable’. These bees might have been more likely to die during pupation or prior to inoculation.

**Table 3.** Effects of nicotine and anabasine consumption on mortality in ‘Stable’ experiment. Table shows marginal significance of individual terms in Cox proportional hazards test for effects of predictor variables on mortality hazard rate.

Source	χ2	Df	P
Nicotine	0.14	1	0.71
Anabasine	0.21	1	0.65
Nicotine*Anabasine	0.19	1	0.66
Colony	7.6	3	0.054

**Table 4.** Effects of nicotine and anabasine on parasite loads in ‘Stable’ experiment. Results of Wald tests for marginal significance of terms in a generalized linear mixed model with penalized quasi-likelihood parameter estimation. “Mass” refers to bee mass at time of emergence.

Source	χ2	Df	P
Nicotine	5.84	1	0.026
Anabasine	2.78	1	0.095
Nicotine*Anabasine	2.59	1	0.11
Colony	6.76	3	0.080
Mass	6.91	1	0.0086

### Consumption experiment

**Figure 5.**
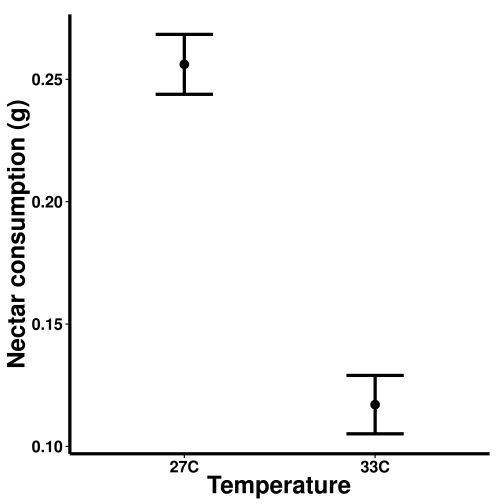
Effects of incubation temperature on nectar consumption in uninfected bees. Points show adjusted mean nectar consumption at each incubation temperature. Error bars represent ±1 standard error. Sample sizes: n=69 (27°C), n=74 (33°C).

Contrary to our expectation that increased temperature would increase nectar consumption, bees drank significantly less when incubated at 33°C than at 27°C ( Table 5). Mass-adjusted mean consumption at 33°C (117 mg) was less than half that at 27°C (256 mg) ( Figure 5). Bee mass was also a significant predictor of consumption, but colony of origin was not ( Table 5).

**Table 5.** Anova results for effects of incubation temperature on nectar consumption at 27°C and 33°C.

Source	SS	Df	F	P
Temperature	0.64	1	64.04	<0.001
Colony	0.01	1	0.98	0.33
Mass	0.24	1	23.61	<0.001
Residuals	1.38	138		

Data for Consumption experiment Day ‘One’ was omitted from analysis due to use of a different style of feeder tube from that used on subsequent days. Abbreviations: Day: day of experiment; Temp: temperature of incubator; Colony: hive of origin; Vial: unique identifier for each sample; Type: “Bee” or evaporation “Control” (i.e., empty vial without a bee); Date: Date of start of trial; Start.time: time at start of trial; Lid.mass.0h: mass of full tube with nectar at start of trial; comment.0h: observations at start time; End.time: clock time on following day at end of trial; Lid.mass.24h: mass of nectar tube after 24h of bee feeding; Bee.mass: mass of bee following the trial; comment.24h: observations at end of trial; consumption.gross: Difference between Lid.mass.0h and Lid.mass.24h; consumption.net: consumption.gross minus average mass loss of bee-free control tubes on same date, which estimates amount of nectar consumed. Incubator: location of trial. Click here for additional data file. Copyright: © 2015 Thorburn LP et al. 2015 Data associated with the article are available under the terms of the Creative Commons Zero "No rights reserved" data waiver (CC0 1.0 Public domain dedication).

## Discussion

### Nicotine increased mortality under variable temperature conditions

Nicotine consumption increased mortality in ‘Variable’, but did not affect mortality in ‘Stable’. The climatic differences between the two experiments may be responsible for this context-dependent response. In ‘Variable’, the incubation temperature of the experimental bees was not controlled: Experimental bees, pupal clumps, and colonies were kept in a room with temperatures that ranged from 10 to 35°C, with the temperature usually at the high end of that range during the day. The stressful nature of the conditions was evidenced the overall higher masses of bees in ‘Variable’, which we believe reflects pre-experiment mortality of many smaller and weaker bees. In ‘Stable’, by contrast, bees were incubated at a constant temperature of 27°C.

We hypothesize that the toxic effects of nicotine in the ‘Variable’ experiment may have been exacerbated by exposure to heat and fluctuating temperatures. Interaction between heat stress and secondary metabolites has been documented in several other species (reviewed in Holmstrup *et al.*, 2010), including some insects and related arthropods. For example, Li *et al.* (2014) found synergistic interaction between heat stress and avermectin toxicity in the western flower thrips ( *Frankliniella occidentalis*), which led to reduced survival and increased upregulation of heat shock proteins. Mercury exposure reduced heat tolerance in springtails ( *Folmosia candidia)* ( Slotsbo *et al.*, 2009), and high temperature increased uptake and toxicity of organophosphate insecticides to the midge *Chironomus tentans* ( Lydy *et al.*, 1999). Our results suggest that interaction between heat stress and toxins may occur in *B. impatiens* as well. An experiment in which temperature and secondary metabolite consumption are manipulated in a factorial design would more definitively test for such interaction.

Our results indicate that nicotine can be toxic to bumble bees even at very low concentrations when bees are parasitized. Another recent study ( Baracchi *et al.*, 2015) also found that chronic consumption of low concentrations (2.5 ppm) of nicotine increased mortality in uninfected *B. terrestris*, although not in those infected with *Crithidia*. These findings contrast with previous studies of honey bees, which did not find significant effects of naturally occurring nicotine concentrations on mortality in bees of unknown parasite status. Detzel & Wink (1993) determined the honey bee LD _50_ for nicotine to be 2000 ppm, far higher than any concentration that occurs in nectar. Singaravelan *et al.* (2006) found that larval survival of honey bees was not affected by naturally occurring concentrations of nicotine (up to 5 ppm), even when consumed consistently for several days, although a much higher concentration of nicotine (50 ppm) did significantly reduce survival. The discrepancy between their results and ours may be due to bumble bees having a greater sensitivity to nicotine than do honey bees. Further study is needed to compare the tolerance of a range of pollinator species to secondary metabolites consumed alone and in combination.

Nicotine had stronger effects on mortality in the ‘Variable’ than in the ‘Stable’ experiment. This result suggests that sensitivity of bumble bees to nicotine is further increased under temperature-stressed conditions that are nonetheless realistic in many habitats. Drastic temperature variation similar to that experienced by bees in ‘Variable’ is common in continental climates, where bumble bees are abundant. For example, in Amherst, MA, where this study was conducted, daily temperature swings of over 15°C are common, and temperatures as low as 10°C and as high as 30°C are frequently experienced within a few days of each other, or even within a single day ( Menne *et al.*, 2012a; Menne *et al.*, 2012b). Wild bees, therefore, are likely to experience temperature conditions under which nicotine could be significantly toxic.

### Under variable temperature conditions, nicotine and anabasine—but not their combination—decreased infection

To our surprise, the ‘Variable’ conditions under which nicotine consumption increased mortality were also the conditions under which alkaloid consumption reduced parasitism. In ‘Variable’, bees that consumed either alkaloid alone had significantly lower parasite counts than control bees; in contrast, bees that consumed both alkaloids had similar parasite counts to controls. Our results are consistent with the results of recent studies that found reduced parasite loads under nicotine and anabasine consumption ( Baracchi *et al.*, 2015; Richardson *et al.*, 2015a). The reduction in parasite load may be due to alkaloid-induced increases in gut motility. Both nicotine and anabasine have been demonstrated to reduce gut transit time in the Palestinian sunbird *Nectarinia osea* ( Tadmor-Melamed *et al.*, 2004). Although their effect on gut transit time in insects has not been studied, rapid excretion is known to be part of some insects’ physiological response to alkaloids ( Wink & Theile, 2002). It is therefore plausible that consumption of nicotine and anabasine could cause an increased rate of excretion in bees, thus clearing *C. bombi* cells from the gut and leading to the observed reduction in parasite load.

The lack of effect of the combined alkaloids on parasite load is more puzzling. The concentrations of the individual alkaloids may have been within the medicinal window of concentration at which antiparasitic effects were dominant. However, the combined effects of both alkaloids may have weakened bees’ ability to fight infection through excessive stimulatory, laxative, and/or immunosuppressive effects. These combined toxic effects could have offset the medicinal effects realized at lower concentrations in the single-alkaloid treatments.

### In a controlled temperature environment, nicotine increased parasite loads without affecting mortality

When bees were kept under constant conditions in ‘Stable’, alkaloid ingestion had different effects on mortality and parasitism from those found in ‘Variable’. In contrast with the antiparasitic effects of alkaloids in ‘Variable’, in ‘Stable’, nicotine consumption significantly increased parasite counts, while anabasine also increased parasite loads, although not significantly. This result is consistent with a growing body of research demonstrating that neonicotinoids, a class of insecticides chemically similar to nicotine, have immunosuppressant effects on bees (reviewed in Goulson *et al.*, 2015). While the effects of nicotine are not necessarily the same as those of neonicotinoids, both nicotine and neonicotinoids function as nAChR agonists, ( Jeschke *et al.*, 2011), suggesting similar pharmacological activity.

The immunosuppressant effects of neonicotinoids have been most well studied in honey bees. Under lab conditions, sub-lethal colony-level exposure to imidacloprid increased levels of *Nosema* infection ( Pettis *et al.*, 2012). In the field, colonies that had foraged on corn treated with thiabendazole had significantly elevated levels of black queen cell virus and *Varroa* mites ( Alburaki *et al.,* 2015). At the molecular level, clothianidin and imidacloprid induced increased transcription of a gene coding for a negative modulator of NF-Kβ immune signaling in honey bees, causing decreased immune function and increased viral replication ( Di Prisco *et al.*, 2013). Additional studies on bumble bees are needed to compare the effects of neonicotinoids and naturally occurring alkaloids, as well as to evaluate how the effects of these compounds vary across species, genera, and dietary contexts.

### Interactive effects of abiotic conditions, alkaloids, and parasites on bees

We hypothesized that the stronger medicinal and toxic effects of the alkaloids in ‘Variable’ may have resulted from increased total alkaloid consumption due to elevated nectar intake under the generally hotter conditions of ‘Variable’. However, our Consumption experiment showed that increasing incubator temperature from that used in ‘Stable’ (27°C) to one characteristic of ‘Variable’ (33°C) significantly *decreased* the volume of nectar consumed. Hence, it appears that the warmer temperatures of ‘Variable’ would have curtailed nectar consumption rather than promoting it. Still, we cannot exclude the possibility that warmth-related decreases in consumption would have been offset by other factors that promoted nectar intake, including increased activity, energy expenditure, and stress-related defecation in the comparatively stimulating experimental environment. In addition, the Consumption experiment used uninfected bees, whereas bees in both ‘Variable’ and ‘Stable’ were inoculated with parasites. Further investigation is needed to define how the thermal environment, external stimuli, nectar alkaloid content, and infection interact to influence patterns of consumption.

The stronger effects of alkaloids in ‘Variable’ may therefore reflect complex interactions between alkaloids, temperature stress, environmental stimuli, and immunity rather than simple differences in alkaloid consumption. Under the fluctuating conditions of ‘Variable’, bees may have been more susceptible to the effects of the alkaloids, both in the form of increased toxicity and increased gut motility, accounting for both the higher mortality and decreased *C. bombi* counts in ‘Variable’. For example, the hot conditions of ‘Variable’ would appear to have increased evaporative moisture losses while decreasing nectar intake, leaving bees more susceptible to dehydration. Nicotine's agonistic effects on intestinal peristalsis may have helped to clear parasites from the gut, but also have contributed to further dehydration. In addition, bees in ‘Variable’ were exposed to external stimuli in the lab environment, including light and vibration, which may have synergized with stimulatory effects of the alkaloids to increase defecation. In the lab, bees often defecate explosively when startled—sometimes tens of centimeters into the air (ECPY, personal observation). Bees on the lab bench in ‘Variable’ would have been agitated more frequently than bees in the incubator in ‘Stable’, and their sensitivity to agitation may have been increased by alkaloid consumption.

The higher temperatures of ‘Variable’ may have additionally functioned as an externally imposed fever that reversed the immunosuppressive effects of nicotine. Febrile amelioration of infection has been shown in many animals (reviewed in Kluger, 1978), including honey bees ( Campbell *et al.*, 2010) and other insects ( Stahlschmidt & Adamo, 2013). The lower absolute parasite counts relative to ‘Stable’ may reflect heat-related inhibition of *C. bombi*, which grows best at 27°C ( Salathé *et al.*, 2012). Stimulatory effects of nicotine and anabasine, enhanced by exposure to everyday disturbance in ‘Variable’, could have increased activity level and metabolic rate, thereby further raising body temperature and slowing parasite growth, albeit at the expense of increasing heat stress on bees. The effects of a given increase in body temperature would have been more pronounced under the hot conditions of ‘Variable’, which may have approached the parasite’s thermal tolerance limit.

Our results in ‘Stable’ contrast with those of a recent study ( Baracchi *et al.*, 2015), which showed that nicotine consumption reduced parasite loads in *Bombus.* There were a number of differences between that study and ours that may explain the differing results. First, the two experiments used different bee species: Baracchi and colleagues used the larger *B. terrestris,* which may be more resistant to potential immunosuppressive effects of nicotine. Even within our experiments, which used bees of a single species from a single supplier, we found highly significant effects of mass and colony, indicating strong effects of both size and genetic variation on parasitism that are consistent with previous findings ( Otterstatter & Thompson, 2006). Interspecific variability could be tested by comparing effects of secondary metabolites on parasitism in a range of *Bombus* species. Second, Baracchi *et al.* employed different bee housing materials. Bees were housed in petri dishes, which may have been less confining than our snap-cap vials, as suggested by the overall lower rates of mortality in the experiments of Baracchi *et al.* compared to ours. This extra space could have allowed bees to take advantage of alkaloid-induced increases in gut motility by expelling *Crithidia*-rich feces in less-frequented parts of their arenas, whereas our bees would have had close and persistent contact with their own waste. It would be intriguing to investigate how the effects of alkaloids differ depending on levels of crowding, including in densely populated hive environments. A third explanation could be that the antiparasitic effects of alkaloids depend on other environmental cues, including daylight. Both our study and that of Baracchi *et al.* showed medicinal effects of nicotine when bees were housed on the lab bench (as in ‘Variable’), whereas nicotine increased parasitism for bees housed in constant darkness in ‘Stable’. Future experiments are needed to explicitly test the roles of environmental conditions and stimuli on bee and parasite tolerance to alkaloids.

Our results also contrast with those of Richardson *et al.* (2015a), which employed the same bee species and unlit rearing conditions as our ‘Stable’ experiment, but found that both nicotine and anabasine significantly reduced *C. bombi* parasite load in *B. impatiens*. The differing effects of nicotine may be due to our use of the (-)-enantiomer of nicotine, whereas Richardson *et al.* (2015a) used a +/- enantiomeric mixture (Sigma N0267, personal communications). (-)-Nicotine, which is far more common in nature ( Armstrong *et al.*, 1998), appears to be more pharmacologically active than (+)-nicotine in vertebrates, aquatic invertebrates ( Barlow & Hamilton, 1965; Gause & Smaragdova, 1939), and insects including honey bees ( Tomizawa & Yamamoto, 1992). Interestingly, Gause & Smaragdova (1939) found the two enantiomers to be isotoxic to protozoa. If (-)-nicotine is more toxic to bees than is (+)-nicotine, but both enantiomers are equally toxic to *C. bombi*, then (-)-nicotine might have lesser medicinal value.

Another possible explanation for our differing results relates to the *C. bombi* itself. *C. bombi* is known to be genetically diverse; Salathé & Schmid-Hempel (2011) identified 213 strains infecting bumble bees in Switzerland. Multiple strains are often present in a single host. Tognazzo *et al.* (2012) found that 67% of infected workers and 54% of infected queens carried mixed-genotype infections, with queens harboring up to 29 different genotypes. In addition, it is possible that not all supposed *C. bombi* infections in fact represent a single *Crithidia* species. Schmid-Hempel & Tognazzo (2010) identified two genetically and morphologically distinct lineages within the *C. bombi* complex, which they classified as cryptic species. They retained the name *C. bombi* for the lineage which more closely matches Lipa & Triggiani’s (1988) original description of *C. bombi*, and proposed the name *C. expoeki* for the other lineage. Both lineages are present in both Europe and North America, suggesting an old divergence. If our *C. bombi* cultures and those used by Richardson *et al.* (2015a) and ( Baracchi *et al.*, 2015) represent different strains, or different species, it is possible that they vary in alkaloid tolerance. In addition, these strains might vary in virulence and pathogenicity. The medicinal effects of alkaloids in ‘Variable’, where absolute parasite loads were lower overall, suggests that alkaloid ingestion might be most therapeutic against relatively mild infections.

### Implications of secondary metabolites for pollinator health in a changing landscape

Our results represent an important first step towards understanding the interactive effects of multiple secondary metabolites on pollinators. We did not find evidence for synergy between *Nicotiana* nectar alkaloids, although we did find some evidence for antagonism. To elucidate the potential role of interactions between compounds in the plant-pollinator-parasite system, it will be necessary to test for interactions between other sets of compounds. Within *Nicotiana*, the wild tobacco *N. attenuata* contains at least 35 nectar secondary compounds, including sesquiterpenes ( Kessler & Baldwin, 2007); many terpenoids have potent trypanocidal activity, yet are relatively benign to animal cells ( Otoguro *et al.*, 2011). Among other plant families, *Asclepias* species are pollinated by bumble bees and contain several cardenolides in their nectar ( Manson *et al.*, 2012) that could be tested for interactive effects. Another plant species to investigate is *Chelone glabra*, which has high concentrations of the iridoid glycosides aucubin and catalpol in its nectar ( Richardson *et al.*, 2015b). Synergy between these glycosides has been demonstrated in their effect on *Junonia coenia* caterpillars ( Richards *et al.*, 2012).

Overall, the effects of nectar alkaloids on parasitized pollinators may represent a tradeoff between toxicity to the parasite and toxicity to the host. In the case of nicotine, bees appear to be more sensitive to alkaloid toxicity than parasites are. While nicotine inhibits the growth of many microbial pathogens, significant antimicrobial effects require concentrations between 100 and 250 ppm ( Pavia *et al.*, 2000). By contrast, Singaravelan *et al.* (2006) found that nicotine was toxic to bees at 50 ppm, and our own results suggest that nicotine can have toxic effects at concentrations as low as 2 ppm. However, the studies establishing toxicity of nicotine in bees have all focused on chronic consumption of a diet high in nicotine. In nature, bumble bees are generalist pollinators, and are known to forage on several plant species within a narrow time frame and even within a single foraging trip ( Free, 1970). In landscapes with varied floral resources, they might be able to avoid chronic nicotine toxicity by exploiting a range of plant species.

## Conclusion

Our results emphasize the importance of interactions between stressors in pollinator health, and demonstrate that the effect of any single factor can vary greatly depending on the other factors involved. Research on pollinator health often focuses on single factors in isolation; however, in natural conditions, pollinators are often exposed to several stressors simultaneously ( Goulson *et al.*, 2015). Previous research has demonstrated both medicinal and toxic effects of secondary metabolites such as nicotine and anabasine. Our results suggest that the predominant effect can vary with environmental and dietary context. In order to better elucidate the role of secondary metabolites in pollinator health, future research should explicitly address the role of these complex interactions.

## Data availability

The data referenced by this article are under copyright with the following copyright statement: Copyright: © 2015 Thorburn LP et al.

Data associated with the article are available under the terms of the Creative Commons Zero "No rights reserved" data waiver (CC0 1.0 Public domain dedication). 

*F1000Research*: Dataset 1. Data for ‘Variable’ experiment, 10.5256/f1000research.6870.d101937 ( Thorburn *et al.*, 2015a).

*F1000Research*: Dataset 2. Data for ‘Stable’ experiment, 10.5256/f1000research.6870.d101940 ( Thorburn *et al.*, 2015b).

*F1000Research*: Dataset 3. Data for Consumption experiment, 10.5256/f1000research.6870.d109062 ( Thorburn *et al.*, 2015c).

## Figures and Tables

**Table S1.** Sample sizes for the 2 experiments, ‘Variable’ and ‘Controlled’.

Treatment	Experiment	
	Variable	Stable
Control	45	83
Nicotine	46	79
Anabasine	46	91
Nicotine + Anabasine	41	86
*Total*	178	339

**Table S2.** GLMM for effects of nicotine and anabasine consumption on mortality in ‘Stable’ experiment. Table shows binomial mixed model results of χ2 tests for effects of predictor variables on probability of death during the 7 d experiment.

Source	χ2	Df	P
Nicotine	0.117	1	0.7323
Anabasine	0.0444	1	0.8331
Nicotine*Anabasine	0.5456	1	0.4601
Colony	6.8536	3	0.0767

## Supplementary material

Script 1. R script for analysis of *C. bombi* parasite loads in ‘Variable’ experiment.

http://dx.doi.org/10.5256/f1000research.6870.s101933

Script 2. R script for analysis of mortality in ‘Variable’ experiment.

http://dx.doi.org/10.5256/f1000research.6870.s101934

Script 3. R script for analysis of *C. bombi* parasite loads in ‘Stable’ experiment.

http://dx.doi.org/10.5256/f1000research.6870.s101935

Script 4. R script for analysis of mortality in ‘Stable’ experiment.

http://dx.doi.org/10.5256/f1000research.6870.s101936

Script 5. R script for analysis of ‘Consumption experiment’.

http://dx.doi.org/10.5256/f1000research.6870.s109063
