# Supplementary material for: Variable effects of nicotine, anabasine, and their interactions on parasitized bumble bees
Source: F1000Res. 2015 Dec 16;4:880. Originally published 2015 Sep 21. [Version 2] doi: 10.12688/f1000research.6870.2 (PMC4786900; doi:10.12688/f1000research.6870.2)
Supplement: Peer review history

# Peer review: Referee response for version 2

**DOI:** 10.5256/f1000research.8151.r11592

**Published:** 2016-01-29

**Authors:** Michael Simone-Finstrom, Margarita Lopez-Uribe

**Affiliations:** 1 Honey Bee Breeding, Genetics, and Physiology Research, USDA Agricultural Research Service, Baton Rouge, LA, USA; 2 Department of Entomology, North Carolina State University, Raleigh, NC, USA

Overall we feel that the authors fully addressed our concerns and those of the other reviewer and significantly improved their manuscript. The fact that they were able to conduct a short experiment adds to the paper and to their conclusions, and we appreciate their willingness and ability to do this.

We just have two minor comments, that likely do not require a revision of the paper, but the authors can decide. Perhaps, due to the openness of the reviews and subsequent replies, this discussion is sufficient. However, if the authors choose to, it would be nice if they make their 'a priori' approach for model selection explicit in the manuscript, and we do not think they should be quite so dismissive of the possible interaction between weight and their treatments. For example, in the authors' response to our earlier comments regarding the possible interaction, they state that other papers either don’t report the interaction or it’s non-significant in the Barrachi paper, which is not an entirely satisfying reason to ignore it here. Additionally, the Barrachi paper did find a significant weight x treatment effect for at least one portion of their experiment. It seems as though this test could at least be attempted, but again we do not think that this necessarily requires a revision of the manuscript.

We have read this submission. We believe that we have an appropriate level of expertise to confirm that it is of an acceptable scientific standard.

## References

[1] BaracchiDBrownMJChittkaL: Behavioural evidence for self-medication in bumblebees?. *F1000Res*.2015;4: 10.12688/f1000research.6262.2 73 10.12688/f1000research.6262.2 25949807PMC4406194

# Peer review: Referee response for version 2

**DOI:** 10.5256/f1000research.8151.r10398

**Published:** 2015-12-29

**Authors:** David Baracchi

**Affiliations:** 1 School of Biological and Chemical Sciences, Queen Mary, University of London, London, UK

The authors responded appropriately to my concerns and other important points raised by Dr.’s Simone-Finstrom and Lopez-Uribe. I appreciated that authors conducted an additional ‘Consumption experiment’ as required. Despite the new results are puzzling and did not provide a clear and definitive picture of the ‘story’ they are well discussed. Overall, I found the manuscript improved and I think that it represents an important starting point for further investigations. Accordingly, I recommend indexing the submitted revised article.

I have read this submission. I believe that I have an appropriate level of expertise to confirm that it is of an acceptable scientific standard.

# Peer review: Referee response for version 1

**DOI:** 10.5256/f1000research.7396.r10860

**Published:** 2015-10-19

**Authors:** David Baracchi

**Affiliations:** 1 School of Biological and Chemical Sciences, Queen Mary, University of London, London, UK

The authors investigated how the consumption of nectar alkaloids, either in isolation or combination, affect survival and pathogen load in a pollinator species. The main goal of the paper is to test the synergistic effects of two alkaloids on bee survival and parasite loads. To address this question the authors used the pollinator *Bombus impatiens*, its common midgut parasite, *Crithidia bombi*, and the natural alkaloids anabasine and nicotine, which are two nectar secondary metabolites produced by *Nicotiana* species. They provided evidence that both alkaloids decreased the parasite loads when bumble bees were kept under variable laboratory conditions. By contrast, they showed that nicotine but not anabasine increased the parasite load when bees were kept under controlled laboratory conditions. The consumption of the alkaloids in combinations did not have any synergistic effect, but rather an antagonistic effect especially under variable rearing conditions.

The authors concluded that the interaction between secondary metabolites are complex and that environmental variables may play an important role in determining the positive or negative impact of the diet on pollinator health.

The study addresses an important question and  provides useful results. There are, however, some points that need clarification and some issues that should be addressed.

**Major concerns:**

 

- In "Methods" the authors stated that " In ‘Variable’, pupal clumps were initially incubated on the lab bench, but were later incubated at 30°C in an incubator....". It is not clear if they used the bees obtained from these initial pupal clumps for the experiments or not. If they excluded them, then there is no need to report this information. If not, authors should discuss the possible implications of this "extra" stress.
- "The overall parasite loads in ‘Controlled’ were much higher, with median parasite loads more than double those observed in "Variable". In my view this point is very important and neglected by the authors and it might partially account for the apparent contradiction between the results of "Variable" and "Controlled" experiments. There are evidences for the existence of a trade-off between alkaloid toxicity to the parasite and toxicity to the host. If the parasite load is too high and bumblebee weakened, the toxicity to the bees might overcome that to the parasite, resulting in even higher parasite loads. On the other hand, if  the parasite load is lower, the costs in consuming the alkaloids might have been counterbalanced by the advantages in slowing the progression of the infection. Authors should discuss this point.
- Do authors have any explanation why bumble bees exposed to 2 ppm nicotine had a so high mortality rate after only 7 days since exposure? For instance, Baracchi *et al* 2015 also found a toxic effect of nicotine, but the detrimental effect on bee lifespan started to be evident only about 20-30 days after the start of the experiment.
- "Variable" and "Controlled" experiments also differed in time of year (the experiments were carried out in February-March and May-July respectively). Season is thus an existing confounding factor that authors should seriously take into account in the discussion.
- Authors suggested that under variable conditions, bees may have consumed more liquid, causing ingestions of more alkaloids accounting for both the higher mortality and decreased parasite load in ‘Variable’. Here I respectfully disagree since I would expect that bees constantly exposed to high temperatures would consume more liquid. It is also possible that bees exposed to high temperatures might have less water in the faeces or might defecate less for suppressing water loss, which may results in higher parasite load. Unfortunately, authors did not provide any information about the overall quantity of sucrose consumed by bumblebees over the experiments. I think that authors should address this point with a simple additional experiment. Measuring for few days the quantity of liquid consumed, and possibly the excretion rate of bees exposed to different temperatures, might really help them to better discuss and clarify their findings.

**Minor points:**

 

- The most novel aspect of the manuscript is the fact that authors investigated the possible interactions between two natural alkaloids. I suggest to mention this aspect in the title.
- In my opinion the first paragraph of the introduction is not necessary.
- The second paragraph of the introduction discusses about  the topic of self-medication and preferential feeding on alkaloid enriched nectars by pollinators and Baracchi *et al.* 2015 should be included in the paragraph since it is really pertinent here.
- The authors dedicated an entire long paragraph on the chemical nature on nicotine used by Richardson *et al.* 2015 and the present manuscript. The paragraph is too long. On top of  that, Richarson *et al.* 2015 and Baracchi *et al *2015 (we amended this information in the paper) used different types of nicotine (the former used a +/- enantiomeric mixture, whereas the latter used (-)-enantiomer of nicotine, as the authors of the present work also did) obtaining the same results. Thus, it is improbable that the chemical composition of the alkaloid is responsible for the differences found. I suggest to reduce or remove this paragraph.  
- For the "Variable" data set authors used the GLMM while for the "Controlled" data set authors used a Cox proportional hazards mixed-effects model. Given that the "Variable" data set does not allow to use a Cox prop hazards mixed-effects model,  I agree with this statistical approach, but nonetheless,  it might be useful if the authors analyze the "Controlled" data also with the GLMM, so that a direct comparison of the two situations would be possible.
- "Variable" and "Controlled"  experiments also differed in the fact that only bees in the "Controlled" were kept with constant darkness, which could have an effect on bee locomotor activity, energy consumption etc.. Authors should mention this in the discussion.

I have read this submission. I believe that I have an appropriate level of expertise to confirm that it is of an acceptable scientific standard, however I have significant reservations, as outlined above.

# Response

**Published:** 2015-12-07

**Authors:** Evan Palmer-Young

**Affiliations:** Univ of Massachusetts, USA

Dear Dr. Baracchi,

Many thanks for your thorough critical review of our article. We feel that the revised version is now improved in response to your comments. We address each point below:

**Reviewer comment:** The study addresses an important question and provides useful results.

**Author Response: **We thank the reviewer for this gracious comment.

**Major concerns:**

**Reviewer Comment: **In "Methods" the authors stated that " In ‘Variable’, pupal clumps were initially incubated on the lab bench, but were later incubated at 30°C in an incubator....". It is not clear if they used the bees obtained from these initial pupal clumps for the experiments or not. If they excluded them, then there is no need to report this information. If not, authors should discuss the possible implications of this "extra" stress.

**Author Response:** We now clarify in “Methods: Rearing Conditions” that we used the bees from all of these pupal clumps. The pupal clumps were only moved to the incubator during the last week of inoculations, 2 days before the emergence of the last bees to be inoculated. Hence, all of the bees in the “Variable” experiment would have been exposed to variable temperatures during pupation. We agree that this additional stress could have affected both survival and immunity, and now mention both of these possibilities in the discussion.

**Reviewer Comment: **"The overall parasite loads in ‘Controlled’ were much higher, with median parasite loads more than double those observed in "Variable". In my view this point is very important and neglected by the authors and it might partially account for the apparent contradiction between the results of "Variable" and "Controlled" experiments. There are evidences for the existence of a trade-off between alkaloid toxicity to the parasite and toxicity to the host. If the parasite load is too high and bumblebee weakened, the toxicity to the bees might overcome that to the parasite, resulting in even higher parasite loads. On the other hand, if the parasite load is lower, the costs in consuming the alkaloids might have been counterbalanced by the advantages in slowing the progression of the infection. 

**Author Response: **Thank you for this suggestion. We have added the idea that alkaloids might be most medicinal against mild infections to the final paragraph of “Discussion: Interactive effects of abiotic conditions, alkaloids, and parasites on bees”.

**Reviewer Comment: **Do authors have any explanation why bumble bees exposed to 2 ppm nicotine had a so high mortality rate after only 7 days since exposure? For instance, Baracchi *et al* 2015 also found a toxic effect of nicotine, but the detrimental effect on bee lifespan started to be evident only about 20-30 days after the start of the experiment.

**Author Response: **In the ‘Variable’ experiment, we believe that the stress from fluctuating temperatures contributed to overall higher levels of mortality and greater susceptibility to alkaloid toxicity than under the “standard laboratory conditions” employed in Baracchi *et al *(2015).

The other reviewers, Dr.’s Simone-Finstrom and Lopez-Uribe, had a similar comment about our high reported rates of mortality in ‘Stable’ in comparison with Richardson *et al. *2015 and Baracchi *et al*. 2015. We duplicate that response here:

It is correct that our study found much higher rates of mortality, even under ‘Stable’ conditions (up to 35% over 7 d), than these two prior papers. However, both of these studies employed different rearing conditions from ours in their experiments measuring survival. Richardson *et al. *(2015) measured mortality in microcolonies (3-bee communities) incubated in 500 mL plastic deli containers, as opposed to the solitary bees in 30 mL snap-cap vials used in our experiments. Baracchi *et al. *used individual bees, but they were housed in petri dishes rather than vials; in addition, whereas Baracchi *et al. *kept bees in the same petri dish throughout the experiment (D. Baracchi, personal communication), we moved our bees to clean vials daily, which may have imposed additional stress resulting from handling. Finally, Baracchi *et al. *used a different strain of *Crithidia*, and also a different bee species ( *B. terrestris*) from the one we used ( *B. impatiens*). Any of these social, environmental and genetic factors may have contributed to the observed differences in mortality.

**Reviewer comment: **"Variable" and "Controlled" experiments also differed in time of year (the experiments were carried out in February-March and May-July respectively). Season is thus an existing confounding factor that authors should seriously take into account in the discussion.

**Author Response: **We are hesitant to speculate on the effects of season, because bee colonies were kept indoors in cardboard boxes and inside closed cabinets, which shielded them from circadian cues. In addition, we do not know enough about the rearing conditions employed by our commercial supplier—e.g. how long bees had been raised in captivity, to what seasonal cues they were exposed-- to offer an informed discussion of this point. However, we do feel that exposure to environmental cues was an important factor within the experiment itself, and have emphasized the possible interactive effects of alkaloid consumption together with daylight and lab stimuli, as astutely pointed out in Dr. Baracchi’s final comment. We mention these interactive effects in the first paragraph of “Discussion: Interactive effects of abiotic conditions, alkaloids, and parasites on bees”, and reiterate their possible importance in the new Discussion paragraph contrasting our results with those of Baracchi *et al.*          

**Reviewer comment: **Authors suggested that under variable conditions, bees may have consumed more liquid, causing ingestions of more alkaloids accounting for both the higher mortality and decreased parasite load in ‘Variable’. Here I respectfully disagree since I would expect that bees constantly exposed to high temperatures would consume more liquid. It is also possible that bees exposed to high temperatures might have less water in the faeces or might defecate less for suppressing water loss, which may results in higher parasite load. Unfortunately, authors did not provide any information about the overall quantity of sucrose consumed by bumblebees over the experiments. I think that authors should address this point with a simple additional experiment. Measuring for few days the quantity of liquid consumed, and possibly the excretion rate of bees exposed to different temperatures, might really help them to better discuss and clarify their findings.

**Author response: **We conducted a follow-up experiment (‘Consumption experiment’, Figure 5) in which we measured nectar consumption at 2 different temperatures, 27 °C and 33 °C. The lower temperature matched that used in the ‘Stable’ experiment (formerly ‘Controlled’), whereas the higher temperature was designed to simulate typical daytime temperatures during ‘Variable’. To our surprise, nectar consumption was *reduced *by over 50% at the higher temperature. Accordingly, we have removed speculation that bees in the generally warmer ‘Variable’ experiment may have consumed greater quantities of nectar and alkaloids, and instead suggest that nicotine might have exacerbated stress from dehydration.

Bees in both temperature treatments were housed in an incubator under dark conditions, similar to those in the ‘Stable’ experiment but lacking the light and other stimuli to which bees were exposed in the ‘Variable’ experiment. The interactive effects of temperature and external stimuli offer an intriguing area for further study, and we encourage such investigation in the Discussion. We thank Dr. Baracchi for suggestions and encouragement with the Consumption experiment.

**Minor points:**

**Reviewer comment: **The most novel aspect of the manuscript is the fact that authors investigated the possible interactions between two natural alkaloids. I suggest to mention this aspect in the title.

**Author response: **We agree. We now mention compound interactions in the title.

**Reviewer comment: **In my opinion the first paragraph of the introduction is not necessary.

**Author response: **This paragraph has been shortened and combined with former second paragraph.

**Reviewer comment: **The second paragraph of the introduction discusses about  the topic of self-medication and preferential feeding on alkaloid enriched nectars by pollinators and Baracchi *et al.* 2015 should be included in the paragraph since it is really pertinent here.

**Author response: **Baracchi *et al. *2015 is now cited in the third paragraph of the introduction that documents medicinal effects of secondary metabolites on pollinators.  

**Reviewer comment: **The authors dedicated an entire long paragraph on the chemical nature on nicotine used by Richardson *et al.*2015 and the present manuscript. The paragraph is too long. On top of  that, Richarson *et al.* 2015 and Baracchi *et al *2015 (we amended this information in the paper) used different types of nicotine (the former used a +/- enantiomeric mixture, whereas the latter used (-)-enantiomer of nicotine, as the authors of the present work also did) obtaining the same results. Thus, it is improbable that the chemical composition of the alkaloid is responsible for the differences found. I suggest to reduce or remove this paragraph.  

**Author response: **This paragraph has been reduced by half, but we felt that it was important to note this difference at some level, given the similarities between our study and that of Richardson *et al. *2015 and the multiple studies demonstrating the disparate effects of the two enantiomers. 

**Reviewer comment:** For the "Variable" data set authors used the GLMM while for the "Controlled" data set authors used a Cox proportional hazards mixed-effects model. Given that the "Variable" data set does not allow to use a Cox prop hazards mixed-effects model,  I agree with this statistical approach, but nonetheless,  it might be useful if the authors analyze the "Controlled" data also with the GLMM, so that a direct comparison of the two situations would be possible. 

**Author response: **This analysis is now given in supplementary Table S2.

**Reviewer comment:** "Variable" and "Controlled"  experiments also differed in the fact that only bees in the "Controlled" were kept with constant darkness, which could have an effect on bee locomotor activity, energy consumption etc.. Authors should mention this in the discussion.

**Author response: **We now comment on this difference in a new Discussion paragraph in which we contrast our results with those of Dr. Baracchi and colleagues.

Thank you again for the thorough review and helpful suggestions.

Sincerely,

Evan Palmer-Young on behalf of the authors

# Peer review: Referee response for version 1

**DOI:** 10.5256/f1000research.7396.r10403

**Published:** 2015-10-07

**Authors:** Michael Simone-Finstrom, Margarita Lopez-Uribe

**Affiliations:** 1 Honey Bee Breeding, Genetics, and Physiology Research, USDA Agricultural Research Service, Baton Rouge, LA, USA; 2 Department of Entomology, North Carolina State University, Raleigh, NC, USA

Thorburn *et al. *describe an experiment where they test the dual effect of two secondary plant compounds on bumble bee mortality and parasite load after infections with the pathogen *Crithidia bombi*. This paper addresses an interesting question that follows up recent findings about the potential use of secondary metabolites of nectar as a self-medication mechanism to reduce parasite infections in bumble bees. However, we found several major points that the authors should address to clarify the results of the study and to better frame it within the context of previous findings.

**Major concerns:**

- We found multiple points of the statistical analysis that were unclear:
  For the ‘controlled’ experiment, the authors say they used a Cox proportional hazards mixed-effect model. However, the risk ratio of death is not reported, which is one of the most informative parameters to quantitatively estimate the difference in survival between the four groups. It is also surprising that there was no significant difference between the survival of the groups. From Fig. 3, it seems that the group in the Nicotine+Anabasine had lower probability of survival.na
  Wouldn’t a model that allows for interactions of treatment, colony and mass be more appropriate?
  Regarding mass and colony, since both terms were highly significant, how does that affect the interpretation of the results? Was there no relationship with mass and treatment? Also did the masses of bees from the 'Variable' experiment differ from those in the 'Controlled' experiment due to the stress they experienced during pupation?
- The authors report a high pre-experiment mortality rate in the “controlled” treatment. Is it possible that the pupae were stressed before starting the experiment and that this condition had a significant effect in the bee survival during the experiment? Even in the control bees, a 60% mortality after 7 days was reported. This numbers seem high compared to other recent studies (Baracchi *et al.* 2015, Richardson *et al* 2015).
- It was interesting to find out that the ‘variable’ treatment generated the most interesting results. However, the conflicting findings of the ‘controlled’ and ‘variable treatments need to better reconcile in the discussion. There also needs to be more information reported regarding how the temperature fluctuated.
- Why do authors think that the results of the ‘variable treatment’ were the product of heat stress and not cold stress, or just stress in general? Similarly the fact that the ‘variable treatment’ was during pupation and not just during the treatment period needs to be addressed along with the implications of that possible effect.
- This is an overall comment about the structure of the introduction and discussion. Even though the text is well written, the structure of these sections does not connect ideas very well. It reads as a list of ideas, rather than one story with a beginning and an end.

**Minor comments:**

- In the abstract, authors state that they tested for ‘synergistic’ effect between two alkaloid compounds. However, results show antagonistic effects between them. Would it be better to just broadly state that the authors are testing for possible interactions between these compounds?
- The 2nd to last paragraph in the introduction. The authors should make the point that this is the first study to look at a combination of any alkaloids, and not just nicotine and anabasine, if that’s the case. It makes this study more significant and should be highlighted.
- In M&M, the subheading ‘diet treatment’ could be confused with a treatment where variable amounts of protein, carbohydrates and lipids were used. Would it be better to call this section ‘Secondary Compound Treatments’?
- On a similar note, the authors may consider changing the names of the treatment groups to “Variable” and “Stable” or something to that effect so there are not two control treatments.
- Was the inoculation done before or after the ‘diet treatment’? If it was done before, please mention this section of the methods in the order in which the experiment took place. This will help the reader with clarity about the process.
- The sample sizes for the parasite load analysis could be in the text or figure, rather than in a supplementary file.
- 2nd paragraph of the discussion. Baracchi *et al.* 2015 found the same result of low levels of nicotine causing mortality, which is left out here.
- The section entitled “In a controlled temperature environment…” starts by seemingly contradicting the previous section without any discussion of that. Also these results need to be reconciled with Baracchi *et al. *2015.
- Should be black queen cell virus (instead of brood queen cell virus)
- The paragraph explaining the difference in chemical composition between the nicotine in Richardson *et al* and this manuscript is too long and it still doesn’t explain why there was no effect on the parasite loads.
- The last sentence of the discussion states that ‘bees would be unlikely to consume enough nicotine from nectar to experience toxic effects’. There is a large body of literature on the effects toxic nectar on bees, how can the authors be certain about that? Particularly given that some nectar is stored within the nest and combined with other nectars.

We have read this submission. We believe that we have an appropriate level of expertise to confirm that it is of an acceptable scientific standard, however we have significant reservations, as outlined above.

# Response

**Published:** 2015-12-07

**Authors:** Evan Palmer-Young

**Affiliations:** Univ of Massachusetts, USA

Dear Dr.’s Simone-Finstrom and Lopez-Uribe,

Many thanks for your careful review of our research. We have revised the manuscript to incorporate your suggestions, and hope that you find the new version improved in content and clarity. We respond to each of your points below:

**Major concerns:**

**Reviewer Comment: **We found multiple points of the statistical analysis that were unclear:

For the ‘controlled’ experiment, the authors say they used a Cox proportional hazards mixed-effect model. However, the risk ratio of death is not reported, which is one of the most informative parameters to quantitatively estimate the difference in survival between the four groups. It is also surprising that there was no significant difference between the survival of the groups. From Fig. 3, it seems that the group in the Nicotine+Anabasine had lower probability of survival.

**Author Response: **The death hazard ratios are now reported in the text, along with results of the pairwise comparison between the Control and Nicotine + Anabasine groups. Although the Nicotine + Anabasine group did have a higher estimated death hazard ratio (1.27 ± 0.22 SE) compared to the control group (0.82 ± 0.16 SE), this difference was not statistically significant (p=0.446 for pairwise comparison).

**Reviewer Comment: **Wouldn’t a model that allows for interactions of treatment, colony and mass be more appropriate?

**Author Response: **We agree that it would have been interesting to investigate a more complex model. However, we were concerned that adding additional terms would prevent meaningful tests of the effects of alkaloid consumption, particularly in the ‘Variable’ experiment, which had a smaller sample size and an unbalanced design due to the high mortality among bees that consumed nicotine. Although the ‘Stable’ experiment had a larger sample size, we chose to analyze it with the same model used for the ‘Variable’ experiment, to facilitate comparison between the two results. Therefore, we formulated our models *a priori *without interaction terms. In previous studies testing the effects of secondary metabolite consumption on *Crithidia *infection, interaction terms were found to be non-significant (e.g. Baracchi *et al. *(2015)) or were not reported (e.g., Richardson *et al. *2015 Proc Biol Sci. 22;282(1803):20142471. doi: 10.1098/rspb.2014.2471; Manson *et al. *2010, Oecologia.62(1):81-9. doi: 10.1007/s00442-009-1431-9).

**Reviewer Comment:** Regarding mass and colony, since both terms were highly significant, how does that affect the interpretation of the results?

**Author Response: **This significance shows that *Crithidia *infection intensity varies by mass and also by colony, which is consistent with the results of previous studies (e.g., Otterstatter & Thompson 2006 Parasitology 133(Pt 6):749–61. http://doi.org/10.1017/S003118200600120X), but does not invalidate our conclusions about the effects of treatment. We have emphasized the effects of mass and colony genotype in the new paragraph of the discussion section about the differences between our study and that of Baracchi and colleagues.

**Reviewer Comment: **Was there no relationship with mass and treatment?

**Author Response: **We apologize for this confusion. Bee mass refers to mass at time of emergence from pupation, before treatments were applied. We now clarify this in Methods: Statistical analysis and in the legends of Tables 2 and 4.

**Reviewer Comment: ** Also did the masses of bees from the 'Variable' experiment differ from those in the 'Controlled' experiment due to the stress they experienced during pupation?

**Author Response: **Yes—somewhat surprisingly, bees in the ‘Variable’ experiment were significantly larger (mean = 0.160 g) than in the ‘Stable’ (formerly ‘Controlled’) experiment (mean = 0.121 g). We expect that this reflects high mortality of the smaller bees in ‘Variable’, which might have been more likely to die during pupation or during the two days between emergence and inoculation. This finding has been added to the section of the Results describing the ‘Stable’ experiment, and is mentioned again in the first paragraph of the Discussion.

**Reviewer Comment: **The authors report a high pre-experiment mortality rate in the “controlled” treatment. Is it possible that the pupae were stressed before starting the experiment and that this condition had a significant effect in the bee survival during the experiment?

**Author Response: **In the ‘Variable’ experiment, pre-experiment mortality was indeed high, and yes, likely impacted survival during the experiment as well. The chronic exposure of colonies, pupae, and adult bees to variable temperatures is now noted in the third paragraph of “Methods: Rearing conditions” and in the first paragraph of the discussion.

**Reviewer Comment: **Even in the control bees, a 60% mortality after 7 days was reported. This numbers seem high compared to other recent studies (Baracchi *et al.* 2015, Richardson *et al* 2015).

**Author Response: **It is correct that our study found much higher rates of mortality, even under ‘Stable’ conditions (up to 35% over 7 d), than these two prior papers. However, both of these studies employed different rearing conditions from ours in their experiments measuring survival. Richardson *et al. *(2015) measured mortality in microcolonies (3-bee communities) incubated in 500 mL plastic deli containers, as opposed to the solitary bees in 30 mL snap-cap vials used in our experiments. Baracchi *et al. *used individual bees, but they were housed in petri dishes rather than vials; in addition, whereas Baracchi *et al. *kept bees in the same petri dish throughout the experiment (D. Baracchi, personal communication), we moved our bees to clean vials daily, which may have imposed additional stress resulting from handling. Finally, Baracchi *et al. *used a different strain of *Crithidia*, and also a different bee species ( *B. terrestris*) from the one we used ( *B. impatiens*). Any of these social, environmental and genetic factors may have contributed to the observed differences in mortality. These differences are described in the new Discussion paragraph contrasting our study with that of Baracchi *et al.*

**Reviewer Comment: **It was interesting to find out that the ‘variable’ treatment generated the most interesting results. However, the conflicting findings of the ‘controlled’ and ‘variable treatments need to better reconcile in the discussion.

**Author Response: **We have added and clarified speculation about the reasons for these differences and their implications in “Discussion: Interactive effects of abiotic conditions, alkaloids, and parasites on bees”, wherein we consider a number of non-exclusive possible explanations for differences in the effects of alkaloids’ effects on mortality and parasitism between ‘Variable’ and ‘Stable’:

(1) Increased consumption: We hypothesized that the stronger effects of alkaloids in ‘Variable’ may have resulted from increased liquid (nectar) intake under hot conditions. We tested this idea in the ‘Consumption experiment’. To our surprise, bees drank substantially less at high temperatures similar to those in ‘Variable’. These results are now reported (Figure 5, Table 5).  

(2) Interactions between multiple stressors:

(a) Mortality: Stronger effects of alkaloid intake on mortality in ‘Variable’ may reflect increased susceptibility to alkaloid toxicity in the presence of multiple stressors, including temperature fluctuation and external disturbance. Given the results of the ‘Consumption experiment’, we now speculate that nicotine may have increased mortality in ‘Variable’ by increasing fecal moisture losses. Because the warmer temperatures of ‘Variable’ appear to have increased evaporative moisture loss while reducing nectar intake, any additional nicotine-induced moisture loss would have increased the risk of lethal dehydration.

(b) Parasitism: By the same logic, stronger effects of alkaloid intake on parasitism in variable temperatures may reflect synergistic effects of alkaloids and external stimuli on locomotor and gut activity. In the new paragraph describing the differences between our study and that of Baracchi *et al. *(2015), we have noted that we both we and Baracchi *et al. *showed antiparasitic effects of nicotine consumption when bees were kept on the lab bench (as they were in ‘Variable’), but not when bees were housed in constant darkness (as in ‘Stable’). 

(3) Inhibitory effects of high temperatures on *Crithidia *compensating for nicotine-induced immunosuppression: We suggest that in ‘Stable’, immunosuppressive effects of nicotine contributed to increased parasite loads. In contrast, in ‘Variable’, stimulatory effects of alkaloid consumption on locomotor activity may have caused bee body temperature to reach or exceed the thermal tolerance limits of the parasite. These febrile effects may have offset any immunosuppressive effects of nicotine.

 (4) In the paragraph describing differences between *Crithidia *strains, we have incorporated an additional suggestion from the other reviewer. Dr. Baracchi suggested that nicotine and anabasine may have their greatest medicinal value at lower levels of infection, such as those observed in ‘Variable’, where median *Crithidia *counts were lower than in ‘Stable’.

**Reviewer Comment: **There also needs to be more information reported regarding how the temperature fluctuated. Why do authors think that the results of the ‘variable treatment’ were the product of heat stress and not cold stress, or just stress in general?

**Author Response: **We agree that the effects seen in ‘Variable’ could reflect both heat and cold stress. However, my personal recollection (ECPY) was that most of the time the lab was very hot, around 35 °C, at least during the day when the experimenters were present. Cold stress seemed to be more occasional, with sporadic morning and weekend periods when the temperature decreased to 10-15 °C due to building steam being shut off for repairs. We would have liked to have kept more careful records, ideally from data loggers, but the extent and duration of the variability in temperature was entirely unexpected, and we did not consistently note the precise conditions. Additional detail about the temperature fluctuation has been added to the first paragraph of Methods: Rearing conditions.

**Reviewer Comment: **Similarly the fact that the ‘variable treatment’ was during pupation and not just during the treatment period needs to be addressed along with the implications of that possible effect.

**Author Response: **We have now noted this additional stress throughout the developmental period in 3 places: in the third paragraph of “Methods: Rearing conditions”, at the end of “Results: ‘Stable’”, and in the first paragraph of the discussion.

**Reviewer Comment: **This is an overall comment about the structure of the introduction and discussion. Even though the text is well written, the structure of these sections does not connect ideas very well. It reads as a list of ideas, rather than one story with a beginning and an end.

**Author Response: **We appreciate this gracious compliment, and have attempted to improve the organization of the introduction, where we have shortened or separated some paragraphs. In the discussion, we have shortened the paragraph contrasting our results with those of Richardson *et al. *(2015), and have elaborated on the differences between ‘Stable’ and ‘Variable’ as well as between our study and previous ones.

**Minor comments:**

**Reviewer Comment:** In the abstract, authors state that they tested for ‘synergistic’ effect between two alkaloid compounds. However, results show antagonistic effects between them. Would it be better to just broadly state that the authors are testing for possible interactions between these compounds?

**Author Response:** We agree and have rephrased the abstract. 

**Reviewer Comment:** The 2nd to last paragraph in the introduction. The authors should make the point that this is the first study to look at a combination of any alkaloids, and not just nicotine and anabasine, if that’s the case. It makes this study more significant and should be highlighted.

**Author Response:** Thank you. We now highlight this unique contribution in the aforementioned paragraph. 

**Reviewer Comment:** In M(aterials&Methods), the subheading ‘diet treatment’ could be confused with a treatment where variable amounts of protein, carbohydrates and lipids were used. Would it be better to call this section ‘Secondary Compound Treatments’?

**Author Response:** We have implemented this suggestion. 

**Reviewer Comment:** On a similar note, the authors may consider changing the names of the treatment groups to “Variable” and “Stable” or something to that effect so there are not two control treatments.

**Author Response:** We apologize that the naming of the “Controlled” experiment was confusing. We have implemented this suggestion and renamed the “Controlled” experiment to “Stable”.

**Reviewer Comment:** Was the inoculation done before or after the ‘diet treatment’? If it was done before, please mention this section of the methods in the order in which the experiment took place. This will help the reader with clarity about the process.

**Author Response:** We apologize for the confusion. The inoculation was performed before the secondary compound treatment began. Bees were fed 30% sucrose nectar without secondary metabolites for 2 days after emergence, then inoculated with *Crithidia*. Diet treatments were implemented immediately post-inoculation. We have reordered this description in the methods section (“Rearing conditions”: final paragraph).

**Reviewer Comment: **The sample sizes for the parasite load analysis could be in the text or figure, rather than in a supplementary file.

**Author response:** We have now listed the sample sizes for each analysis in the legends of the relevant figures.

**Reviewer Comment:** 2nd paragraph of the discussion. Baracchi *et al.* 2015 found the same result of low levels of nicotine causing mortality, which is left out here.

**Author response:** This result is now cited (third paragraph of Discussion).

**Reviewer Comment:** The section entitled “In a controlled temperature environment…” starts by seemingly contradicting the previous section without any discussion of that.

**Author response:** We have added two transition sentences at the beginning of this paragraph.

**Reviewer Comment:** Also these results need to be reconciled with Baracchi *et al. *2015.

**Author response:** We have added a full paragraph to the discussion section outlining the differences between our experiments and those of Baracchi *et al. *(2015).

**Reviewer Comment:** Should be black queen cell virus (instead of brood queen cell virus)

**Author response:** Thank you for pointing out this error. It is now corrected. 

**Reviewer Comment:** The paragraph explaining the difference in chemical composition between the nicotine in Richardson *et al* and this manuscript is too long and it still doesn’t explain why there was no effect on the parasite loads.

**Author response:** This paragraph has been substantially reduced, but is retained in a shortened form; the editors encouraged us to include this paragraph in order to reconcile our results with those of Richardson *et al.*, which included experiments done in our own laboratory.

**Reviewer Comment:** The last sentence of the discussion states that ‘bees would be unlikely to consume enough nicotine from nectar to experience toxic effects’. There is a large body of literature on the effects toxic nectar on bees, how can the authors be certain about that? Particularly given that some nectar is stored within the nest and combined with other nectars.

**Author response:** We have now reduced this speculation to say that in varied landscapes, bees might be able to reduce or avoid toxicity by exploiting different, lower-nicotine flower species.

Thanks you again for your critical review and thoughtful suggestions.

Sincerely,

Evan Palmer-Young on behalf of the authors
